# Effect of circulation on the disposition and ocular tissue distribution of 20 nm nanoparticles after periocular administration

**Published:** 2008-01-29

**Authors:** Aniruddha C. Amrite, Henry F. Edelhauser, Swita R. Singh, Uday B. Kompella

**Affiliations:** 1 Department of Pharmaceutical Sciences and; 2 Department of Ophthalmology and Visual Sciences, University of Nebraska Medical Center, Omaha, NE 68198-5840; 3 Emory Eye Center, Emory University, Atlanta, GA.

## Abstract

**Purpose:**

Our previous studies indicated that while 20 nm particles are rapidly cleared from the periocular space of the rat following posterior subconjunctival injection, 200 nm particles persisted for at least two months. To understand faster clearance of 20 nm particles, the purpose of this study was to determine transscleral permeability and in vivo disposition in the presence and absence of circulation. Further, it was the purpose of this study to simulate sustained retinal drug delivery after periocular administration of rapidly cleared and slowly cleared nanoparticles.

**Methods:**

The permeability of 20 and 200 nm particles over 24 h was examined across isolated bovine sclera and sclera-choroid-RPE with or without a surfactant (Tween 20, 0.1% w/v) added to the preparation. The in vivo disposition of nanoparticles was performed using Sprague Dawley rats. The rats, either dead or alive, were administered with 400 µg of the nanoparticles in the periocular space, and the particle disposition in the eye tissues was assessed 6 h later. To evaluate the role of the reticulo-endothelial system and lymphatic circulation, isolated liver, spleen, and cervical, axillary, and mesenteric lymph nodes were analyzed using confocal microscopy. Mathematical simulations with Berkeley Madonna were used to evaluate the effect of nanoparticle size on retinal drug levels following periocular administration. Celecoxib was used as the model drug and the finalized pharmacokinetic model from a previous study was used with some modifications for the simulation.

**Results:**

Transport of 20 nm particles across sclera in the presence and absence of the surfactant were 0.1%±0.07% and 0.46%±0.06%, respectively. These particles did not permeate across the sclera-choroid-RPE in 24 h. There was no quantifiable transport for 200 nm particles across the sclera or the sclera-choroid-RPE. In live animals, the 20 nm particles were undetectable in any of the ocular tissues except in the sclera-choroid following periocular administration; however, in dead animals, the particle concentrations in the sclera-choroid were 19 fold higher than those in live animals, and particles were detectable in the retina as well as vitreous. The retention of 20 nm particles at the site of administration was two fold higher in the dead animals. In live animals, the particles were clearly detectable in the spleen and to a very low extent in the liver as well. The particles were also detected in the cervical, axillary, and mesenteric lymph nodes of the live animals. Simulations with two particles (20 nm and 200 nm) with different clearance rates demonstrated that the retinal drug levels were affected by particle clearance. Larger nanoparticles sustained retinal drug delivery better than smaller nanoparticles. With an increase in drug release rate from the particles, these differences diminish.

**Conclusions:**

The 20 nm particles are transported across the sclera to a minor degree; however, there is no significant transport across the sclera-choroid-RPE. Periocular circulation (blood and lymphatic) plays an important role in the clearance of the 20 nm particles. The higher particle levels in the ocular tissues in the post-mortem studies indicate a dynamic physiologic barrier to the entry of particles into the ocular tissues after periocular administration. The particle size of the delivery system can play an important role in the observed retinal drug levels after periocular administration. Slow release nanoparticles with low clearance by blood and lymphatic circulations are suitable for prolonged transscleral drug delivery to the back of the eye.

## Introduction

The periocular (transscleral) route of delivery is gaining importance as an alternative to the intravitreal administration for drug delivery to the choroid, retina, and vitreous [1]. It has been demonstrated that the transscleral route can deliver therapeutically effective drug levels for the treatment of choroidal neovascularization associated with age-related macular degeneration [2,3]. It has also been demonstrated that the periocular (transscleral) route can be used for delivery of large molecules to the choroid and the retina in vivo [4,5].

Most of the posterior segment disorders like diabetic retinopathy and age-related macular degeneration are of a chronic nature and would require long-term therapy for their treatment. Sustained drug delivery systems can provide sustained drug levels to a particular tissue thereby significantly reducing the dosing frequency and the associated complications. Several delivery systems including implants, scleral plugs, and micro- and nanoparticles have been used for this purpose [6-10]. Among these systems, the particulate systems offer several advantages including ease of repeated injections and biodegradability. In addition, nanoparticles have the advantage of cellular entry, which can be used in the delivery of macromolecules like DNA and proteins inside the cells [11]. The right combination of the drug/polymer can control the rate of delivery of a particular drug from these systems and extend the duration for which the drug is delivered.

Sustained drug delivery to the retina by the periocular (transscleral) route would require that the delivery system be retained at the periocular site for a prolonged time so that the drug can be released from the device and become available to the retina. In the case of gene delivery, it would be ideal if the system penetrates the sclera and enters into the choroid and retina where the cells in the choroid or the retina can be transfected. Thus, based on clinical need, the choice of a particulate delivery system can be made.

We have previously demonstrated that the disposition of particles after periocular administration is size dependent. Specifically, particles in the range of 200–2000 nm were almost completely retained at the site of administration for at least two months while the smaller 20 nm particles were cleared rapidly from the site of administration. We also observed that the intra-ocular tissues like the retina and the vitreous did not have any quantifiable uptake of the particles of any size [12]. In this study, we investigated the probable mechanisms for the rapid clearance of 20 nm particles from the periocular tissue. We also examined the limiting role of static and dynamic physiologic barriers [13] to the entry of the nanoparticles into the intra-ocular tissues after periocular administration. We used in vitro permeability studies to determine whether nanoparticles can cross the sclera to gain access to the choroid and RPE and whether nanoparticles can cross the choroid-RPE to gain access to the neural retina. To understand the limiting nature of dynamic physiologic barriers or circulatory systems, we examined disposition of 20 nm particles after periocular (posterior subconjunctival) administration in live and dead Sprague Dawley (SD) rats.

Microparticles and nanoparticles are useful sustained release drug delivery systems. Based on our investigations, the particle size of the drug delivery system can not only influence drug release rates [14] but also particle clearance [12], which means retinal drug delivery. Thus, another objective of this study was to simulate drug levels in the retina with administration of sustained drug delivery particulate systems of different particle sizes for a model drug, celecoxib, for which we recently developed a periocular pharmacokinetic model [15]. In this study, we simulated sustained retinal delivery of celecoxib, exhibiting different periocular clearance rates, following administration of the drug containing nanoparticles of two different sizes (20 and 200 nm).

## Methods

### Materials

Carboxylate modified polystyrene, bodipy-loaded Fluospheres (20 nm and 200 nm) were obtained from Invitrogen (Carlsbad, CA). Sodium pentobarbital was purchased from Fort Dodge laboratories (Fort Dodge, IA). All other chemicals were of analytical grade and purchased from Sigma (St. Louis, MO).

**Figure 1:**
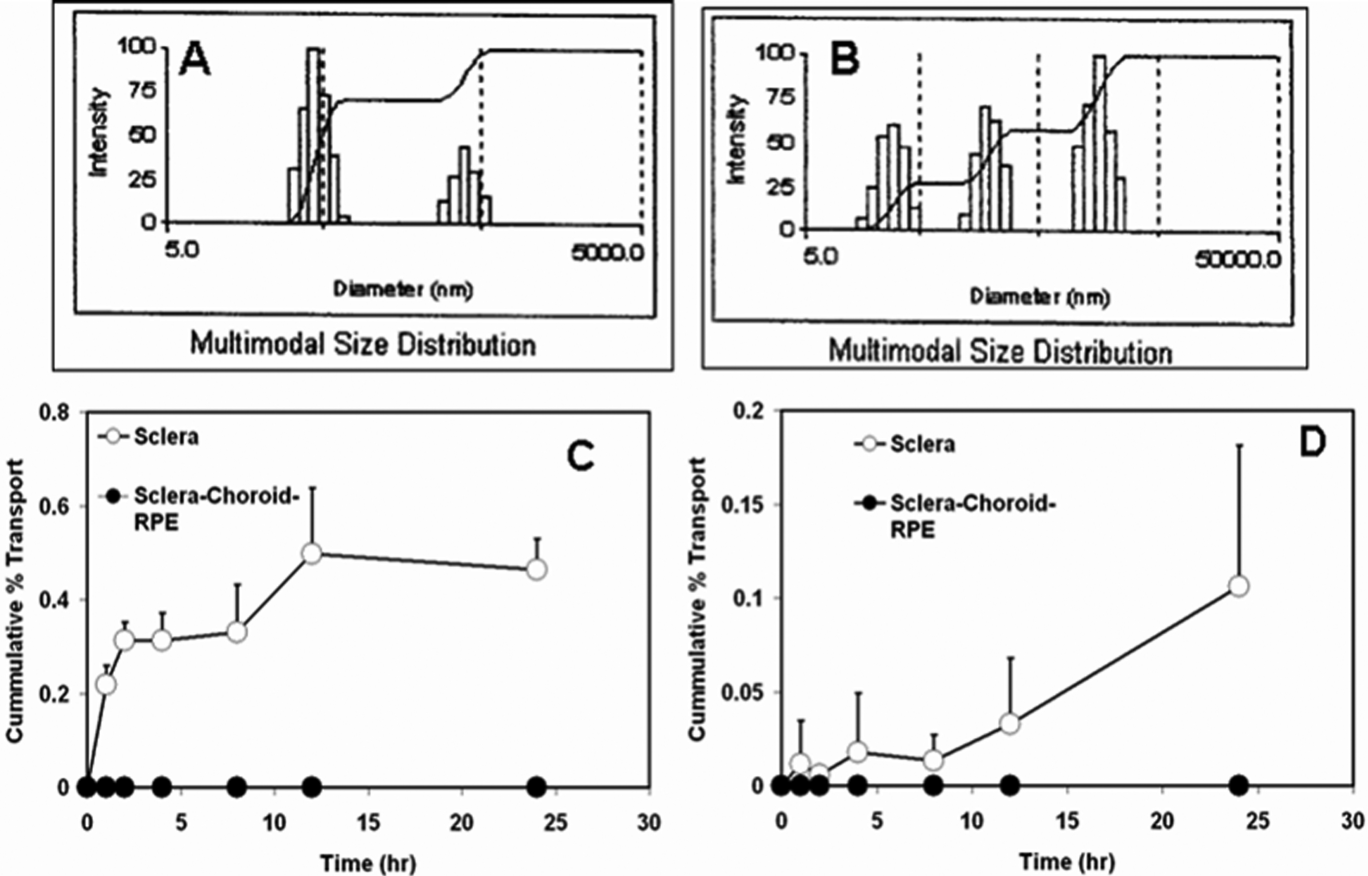
Nanoparticle size distribution and permeability across sclera and sclera-choroid-RPE. **A**: Particle size distribution after 24 h storage in assay buffer used for transport studies. Majority of the particles are distributed around the 50 nm particle size with another small group of particles of higher size distribution. **B**: Particle size distribution after 24 h storage in assay buffer containing 0.1% tween-20. In the presence of the surfactant, the particle size distribution shifts towards greater particle size indicating probable particle aggregation. **C**: Transport of nanoparticles (20 nm; 100 µg/ml) across isolated bovine sclera and sclera-choroid-RPE. The 20 nm particles can cross the sclera but not the sclera-choroid-RPE combination to any quantifiable extent. **D**: Transport of nanoparticles (20 nm; 100 µg/ml) across isolated bovine sclera and sclera-choroid-RPE in the presence of 0.1% tween-20. The particle transport across sclera is reduced in the presence of surfactant probably due to the shift in particle size distribution. Data are expressed as mean ± s.d. for n = 5-6. No quantifiable transport was observed either across the sclera or the sclera-choroid-RPE with the 200 nm particles.

**Figure 2:**
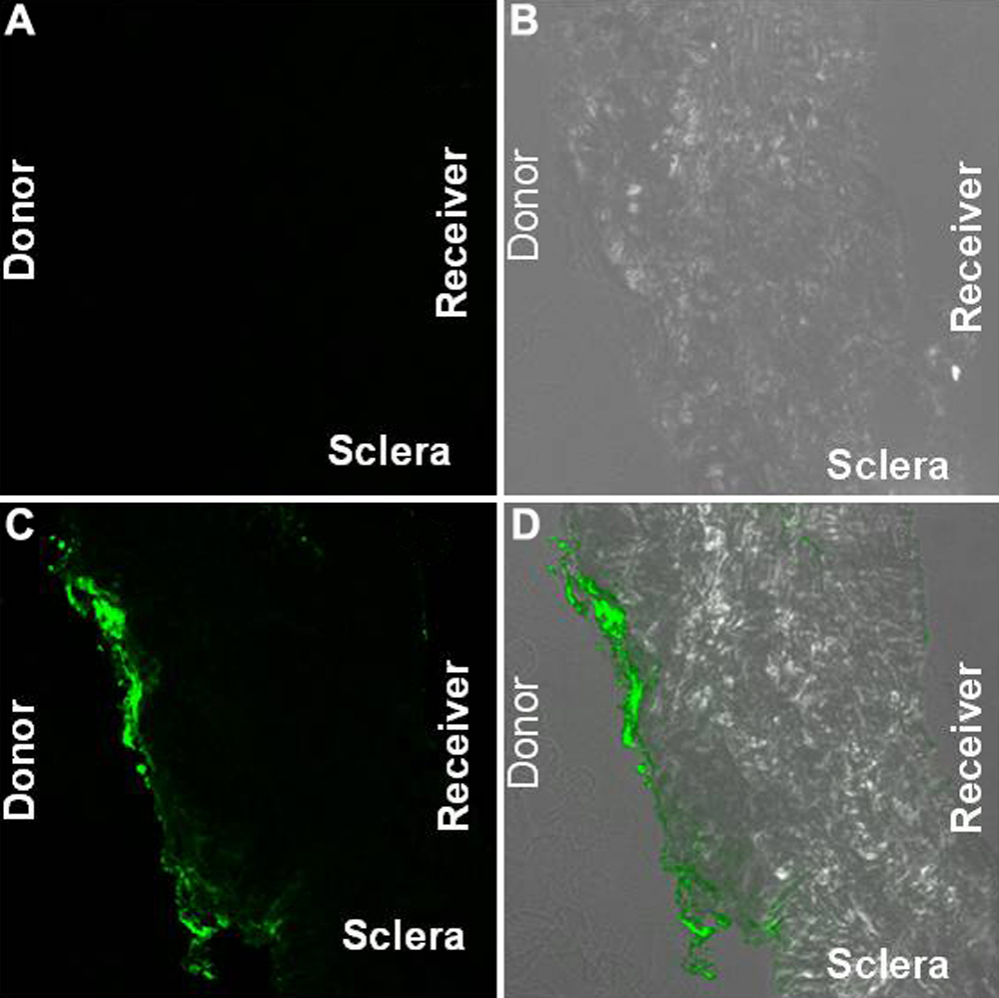
Confocal images of the sclera at the end of the 24 h nanoparticle (20 nm) transport study. **A** shows the control fluorescence and combined fluorescence while **B **shows the phase contrast images. **C** and **D** are nanoparticle exposed tissue fluorescence and combined fluorescence and phase contrast images, respectively. In each image, the scleral (donor) side is on the left and the vitreal (receiver) side is on the right. The particles are concentrated on the outer edge of the sclera. There are very few or no particles on the vitreal side of the tissue.

### Particle size analysis

Particle size analysis was performed by dynamic light scattering as previously described [12].

### In vitro permeability of nanoparticles

The in vitro permeability studies were performed across sclera and the sclera-choroid-RPE isolated from bovine eye as previously described [16]. Bovine eyes were obtained from Nebraska Beef (Omaha, NE). The eyes were kept on ice until dissection. The total time elapsed between the death of the animals and the start of the experiment was less than 4 h in this study. The eye was dissected around the limbus, and the anterior tissues were removed. The eye was then cut along the geometric axis then the vitreous and neural retina were separated. For the sclera-choroid-RPE combination, the tissue required no further processing. To isolate the sclera, the choroid-RPE layer was gently scraped off. The isolated tissues were mounted in modified Ussing chambers with the scleral surface facing the donor side and the vitreal surface facing the receiver side. The bathing fluids were bubbled with 5% CO_2_, 20% O_2_, and 75% N_2_. The donor chamber contained 1.5 ml of 100 µg/ml solution of Fluospheres (20 nm or 200 nm). At several time points up to 24 h, 200 µl of the medium was removed from the receiver chamber and replaced with equivalent assay buffer. The samples were analyzed using a fluorescence plate reader (SpectraMax Gemini; Molecular Devices Corporation, Sunnyvale, CA). To study the effect of surfactant on particle transport, the same study was performed in the presence of 0.1% Tween 20 (a non-ionic surfactant).

To visualize the transport and diffusion of nanoparticles through the tissue, confocal microscopy was used. After performing the transport across the tissues as described above, the part of the tissue exposed to the nanoparticle suspension was cut off from the rest of the tissue and embedded in an optimal cutting temperature (OCT) medium for frozen sectioning. The eyes were kept at −80 °C before sectioning. Sections around 7 µm in thickness were cut and visualized using a Zeiss confocal laser scanning microscope (Carl-Zeiss microimaging Inc., Thornwood, NY).

**Figure 3:**
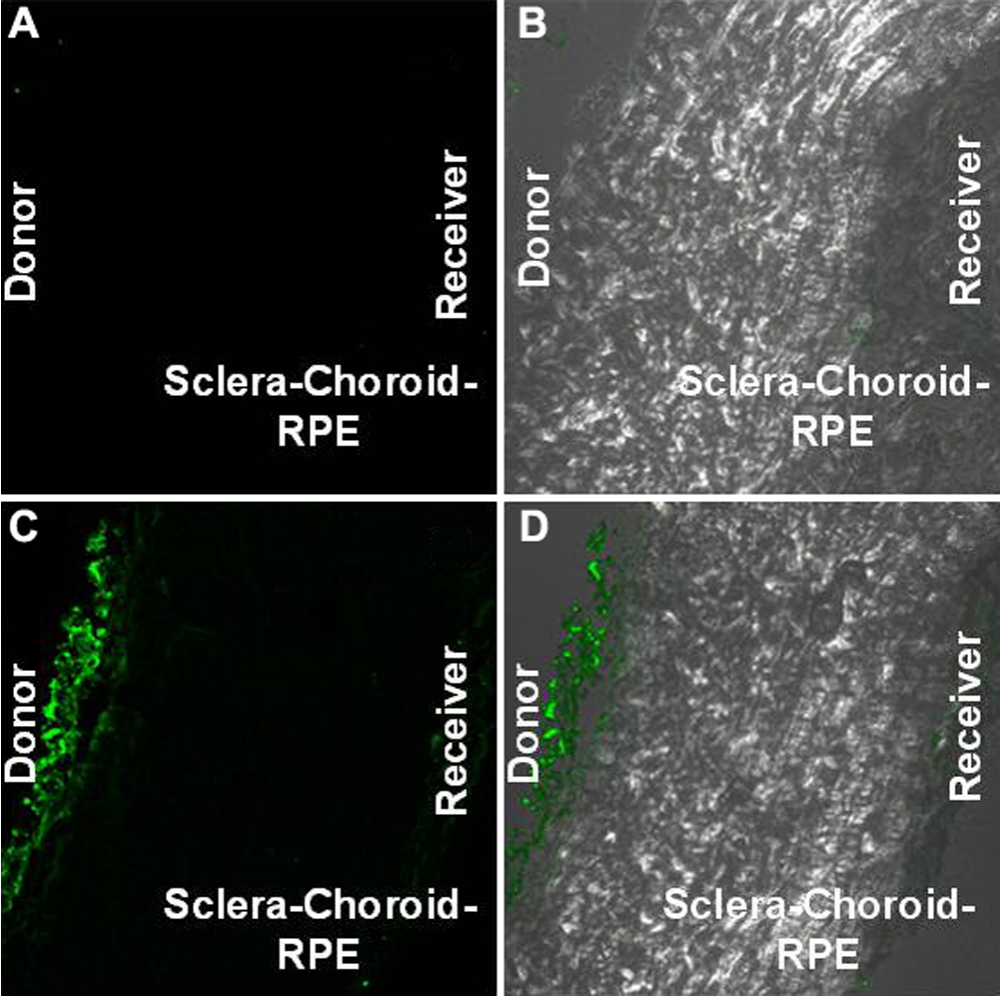
Confocal images of the sclera-choroid-RPE combination at the end of 24 h nanoparticle (20 nm) transport study. Panel **A** shows the fluorescence image and Panel **B** shows the combination (fluorescence plus phase contrast) image of the control sclera-choroid-RPE tissue. Panel **C** shows the fluorescence and Panel **D** shows the combination (fluorescence plus phase contrast) image of the sclera-choroid-RPE tissue that was exposed to nanoparticles during the transport study. In each panel, the scleral (donor) side is on the left and the vitreal (receiver) side is on the right. The particles are concentrated on the outer edge of the sclera. There are very few or no particles seen on the vitreal side of the tissue.

### Effect of circulation on the disposition of 20 nm particles

Carboxylate-modified 20 nm particles (400 µg suspended in 20 µl deionized water) were administered to live or dead Sprague Dawley (SD) rats by periocular administration into the posterior subconjunctival space using a 30G needle. Particle uptake in the ocular tissues as well as the retention of the particles at the periocular site (the tissues in this area include the subconjunctival fat and muscle as well as the conjunctiva) was investigated 6 h after the administration. Since the dead animals were devoid of any circulation and lymphatic flow unlike live animals, the effect of circulation and lymphatics on the disposition of the nanoparticles could be analyzed. All the animal experiments were performed in compliance with the ARVO Statement for the Use of Animals in Ophthalmic and Vision Research.

**Figure 4:**
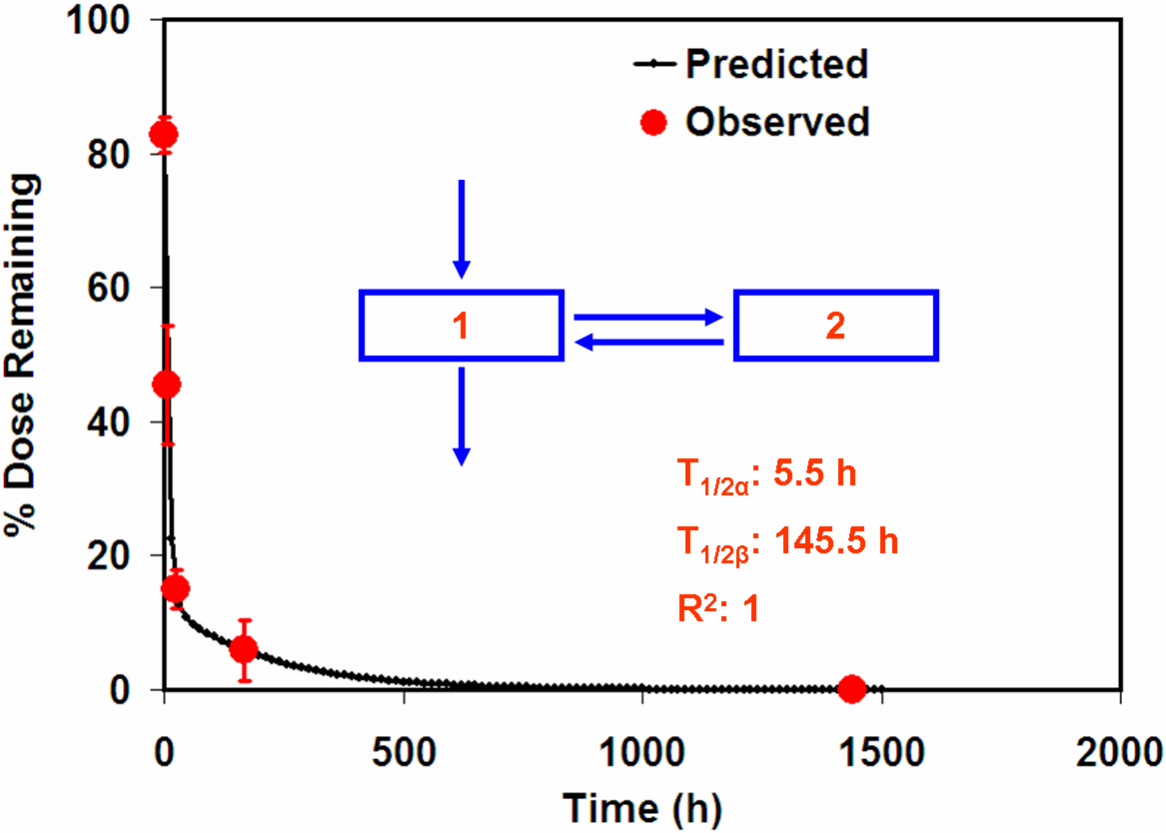
Pharmacokinetic modeling of the disposition of 20 nm particles in the periocular space. Nanoparticle (20 nm) elimination from the periocular tissue is biphasic. The solid line represents the model predicted data while the circles represent the observed data. T_1/2α_ and T_1/2β_ represent half-lives for elimination from the periocular space. R^2^: regression coefficient for the correlation between observed and predicted data.

### Tissue isolation and quantification of intact particles

Tissue isolation and quantification of particles in the ocular and periocular tissues were performed as previously described [12]. Briefly, the ocular tissues, including the sclera-choroid, retina, vitreous, lens, and cornea, were isolated from the frozen eye. The tissues were homogenized in 1 ml of phosphate buffered saline and incubated for 3 h after the addition of 1 ml of 2% Triton-X solution. The fluorescence intensity was measured at the appropriate excitation and emission wavelengths. The periocular tissues (including the subconjunctival fat and muscle as well as the conjunctiva) were homogenized and incubated for 3 h in 1 ml of 2% Triton-X. The incubation mixture was ultrasonicated at 1.5 W for 3–5 min, and the tissue and supernatant were separated by centrifugation. The supernatant was diluted appropriately, and the fluorescence was measured using a Shimadzu RF 5000 spectrofluorometer at the appropriate excitation and emission wavelengths.

**Figure 5:**
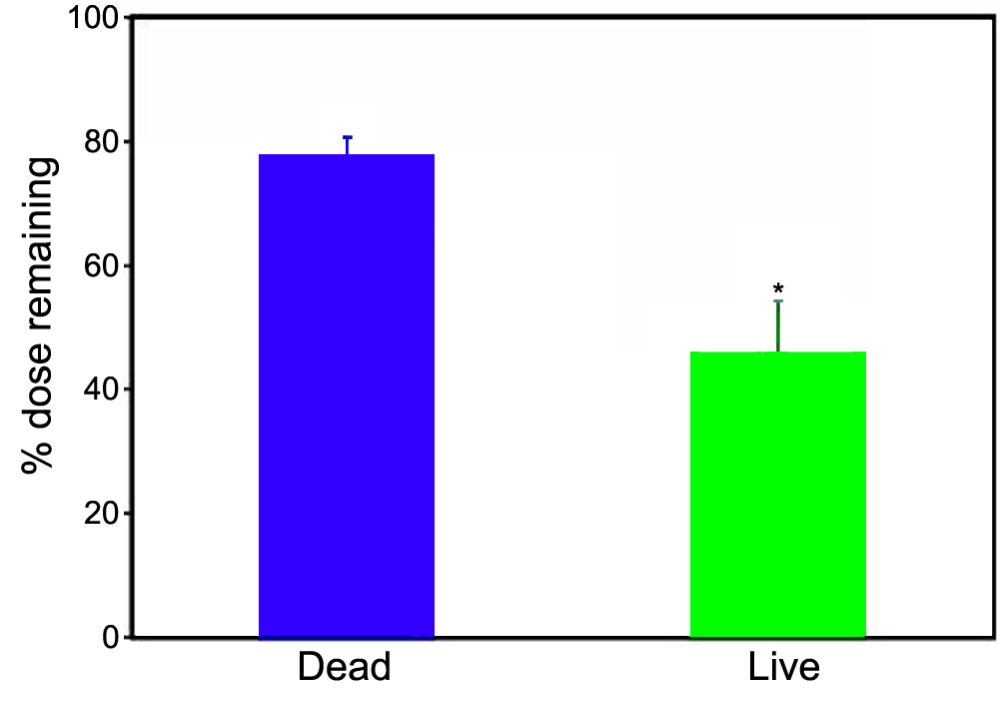
Nanoparticles (20 nm) are cleared by ocular (blood and lymphatic) circulation. Following periocular administration of 400 µg dose of 20 nm particles to either live (blood and lymphatic circulation present) or dead rats (blood and lymphatic circulation absent), the percent dose remaining at the site of administration was determined 6 h post-dosing. The amount of particles remaining in the periocular tissue was more than 2 fold higher in the dead rats as compared to the live rats. The data are expressed as mean ± SEM for n=4. The asterisk indicates a statistically significant difference between live and dead animals (p<0.05).

### Confocal microscopy to study the disposition of 20 nm particles

To analyze the mechanism of disposition of the 20 nm particles, the particles were administered to the live SD rats by periocular injection followed by sacrifice at 6 h. The eye, the periocular tissue, the liver, and the spleen (organs of the reticulo-endothelial system or RES) were isolated and immediately embedded in an OCT medium, and frozen blocks were prepared. Sections (7 µm) were cut from these blocks and analyzed by confocal microscopy to detect the presence of particles in these tissues.

### Analysis of nanoparticles in lymph nodes after periocular injection

SD rats, live and dead, were injected in the posterior subconjunctival region with 400 µg dose of 20 nm nanoparticles in the right eye. Rats, who did not receive an injection, were treated as controls for tissue autofluorescence. The rats were euthanized 6 h after nanoparticle administration. Cervical, axillary, and mesenteric lymph nodes were isolated and immediately fixed in 4% paraformaldehyde overnight. Fixed nodes were embedded in paraffin and sectioned to obtain 10 µ-thick sections. Unstained tissue sections were observed under a confocal microscope at 63x magnification.

### Statistics

Statistical comparison between the dead and the live groups were done by using the non- parametric Mann–Whitney test using SPSS 11.0 (Chicago, IL).

**Figure 6:**
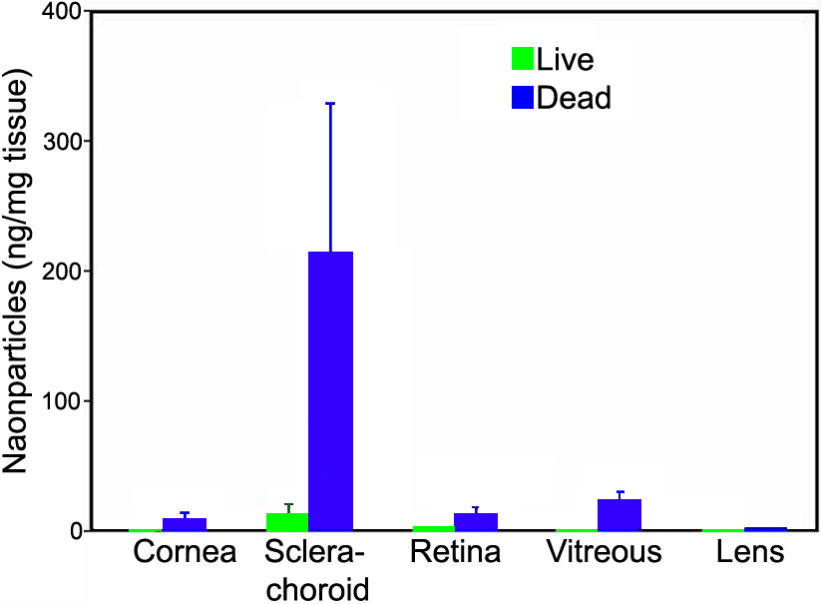
Dynamic barriers prevent significant entry of 20 nm particles into ocular tissues in live animals. Following periocular administration of 400 µg dose of 20 nm particles to either live (blood and lymphatic circulation present) or dead rats (blood and lymphatic circulation absent) the particle levels in the ocular tissues were quantified. Higher levels of the particles are seen in the sclera-choroid, retina, vitreous, and the cornea of dead rats as compared to live rats. The data are expressed as mean ± SEM for n=4.

### Modeling the disposition of the nanoparticles from the periocular tissue

The data from this study (6 h in live animals) after periocular administration of the 20 nm particles and a previous study [12] with time points of 1 day, 7, 15, and 60 days were combined to develop a pharmacokinetic model to describe the disposition of small nanoparticles from the periocular site of administration. The data were fit to a simple one-compartment or two-compartment open model (intravenous bolus administration) from the existing model library of WinNonlin 1.5 (Pharsight Corporation, Mountain View, CA). The best fit model was selected on the basis of visual data fits and statistical goodness of fit metrics including the coefficient of determination and the Akaike information criteria (AIC). All the kinetic parameters were estimated by WinNonlin.

### Simulations of retinal drug levels from sustained release particulate systems of celecoxib with different particle sizes

The finalized model from our previous study, describing the pharmacokinetics of celecoxib in the retina after periocular administration [15], was used as the base model in this study. The previous modeling was based on studies where celecoxib was administered as a suspension, and the elimination of the formulation itself was considered negligible [15,17,18]. However, if a nanoparticulate sustained drug delivery system of celecoxib is used for retinal drug delivery after periocular administration, the nanoparticulate systems can have differential elimination kinetics based on the particle size [12]. Hence, we included an elimination term for the formulation after periocular administration. The half-life for the removal of 20 nm particles from the periocular space was assumed to be 5.5 h. The elimination half-life of 200 nm particles was conservatively assumed to be 180 days (we had observed almost complete retention of the 200 nm particles after periocular administration for a period of two months [12]). Drug levels in the retina were simulated using different release rates of the drug from the particulate system. All the other parameters for the model were obtained from the previous study [15]. All simulations were performed using Berkeley Madonna (Berkeley, CA).

**Figure 7:**
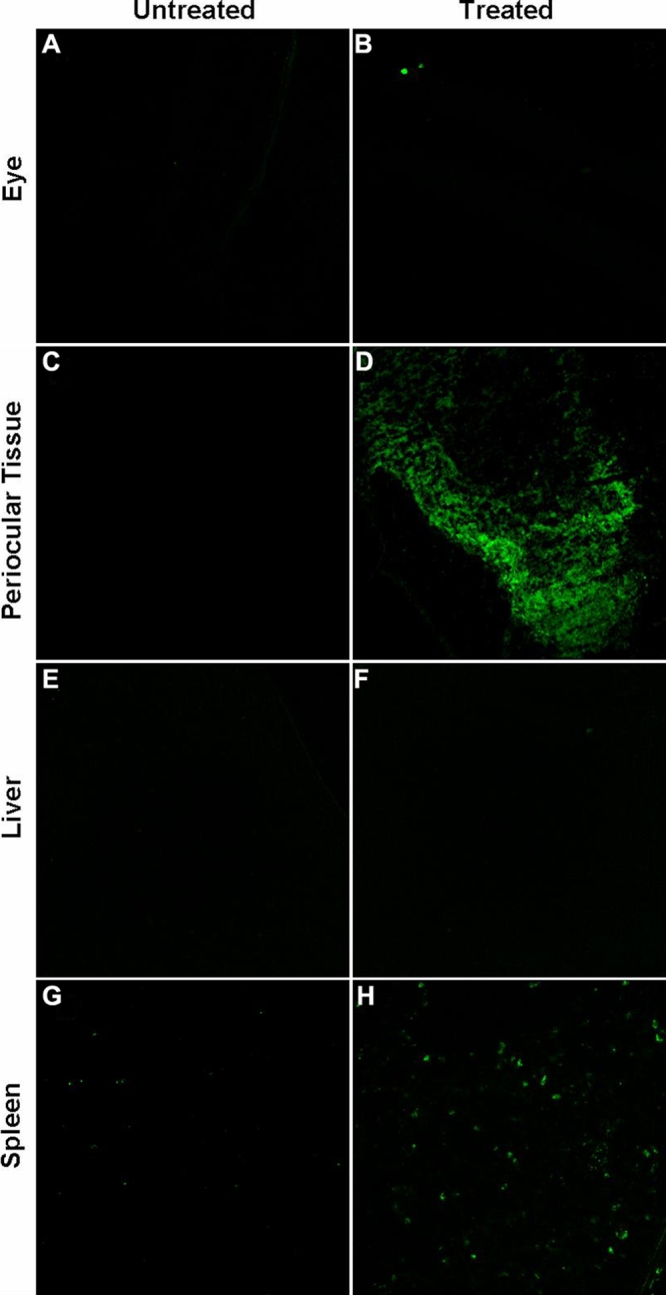
Representative confocal micrographs of various tissues 6 h after periocular administration of 20 nm particles. Following periocular administration of 400 µg dose of 20 nm particles to live rats, the nanoparticles can be found in the organs of the reticulo-endothelial system (liver and spleen). The various tissues including the eye, the periocular tissue, the liver and the spleen were removed and sectioned 6 h after administration. The figure shows the fluorescence images of sections of the: eye (Panels **A** and **B**); periocular tissue (Panels **C** and **D**); liver (Panels **E** and **F**); and spleen (Panels **G** and **H**). The left panels (**A**, **C**, **E**, and **G**) are fluorescence images from control rats that were not dosed with the nanoparticles whereas the right panels (**B**, **D**, **F**, and **H**) are images from the rats that were dosed with the nanoparticles. Nanoaprticles can be seen in the periocular tissue, spleen and to some extent in the liver of the dosed animals.

## Results

### In vitro transport of nanoparticles across isolated bovine sclera and the sclera-choroid-RPE

The mean hydrodynamic diameter of the particles measured by dynamic light scattering was 45 and 230 nm, respectively, for the smaller and larger Fluospheres. The smaller nanoparticles permeated across the sclera with the percentage transport at the end of 24 h being 0.46%± 0.064% (Figure 1). No detectable transport was seen across the sclera-choroid-RPE combination. There was no detectable transport of the 200 nm particles across either sclera or sclera-choroid-RPE. Confocal microscopy revealed that most of the 20 nm particles were concentrated at the edge of the sclera facing the donor chamber (Figures 2 and 3). A few particles could be seen in the deeper sclera and at the other edge. With the surfactant present, the particle transport decreased to 0.1%±0.07% (Figure 1C). This decrease was likely due to the particle aggregation caused by the surfactant as the measured mean hydrodynamic diameter of particles in the surfactant preparation was around 253 nm (Figure 1A,B).

### Pharmacokinetic modeling of particle disposition after periocular administration

Using the data from the current and previous study, a model to describe the pharmacokinetics of the periocularly administered particles was developed using WinNonlin. The model fits to the data were done using the one and two-compartmental models (intravenous bolus models in WinNonlin). Based on visual observations and the goodness of fit metrics, the two-compartmental model was selected to describe the kinetics of disposition in the periocular tissue. The model predicted and the observed data are shown in Figure 4. The particles showed a biphasic disposition with half-lives of 5.5 h and 146 h, respectively, for the two phases.

### Influence of circulation on the disposition of 20 nm particles

Circulation played a critical role in the disposition of 20 nm particles. The retention in the periocular tissue at the end of 6 h of administration in the live animals was 45%±9% whereas the retention in the dead animals was 77%±3% (Figure 5). The sclera-choroid tissue levels in the live and dead animals at the end of 6 h were 12±16 ng/mg and 214±239 ng/mg, respectively (Figure 6). The particles could not be quantified in the cornea, retina, and the vitreous of live animals; however, the particle levels in the cornea, retina, and the vitreous of the dead animals were 9±8 ng/mg, 13±9 ng/mg, and 22±14 ng/mg, respectively (Figure 6). In both the live and dead animals, the particle levels were below quantification limits in the lens.

### Distribution of 20 nm particles after periocular administration using confocal microscopy

The systemic distribution of 20 nm particles in live rats was analyzed by confocal microscopy. The particles were seen in the periocular tissues and to some extent in the spleen. No particles were detected in the ocular tissues, and only a faint fluorescence was found in the liver 6 h post-administration of the nanoparticles to the rats (Figure 7).

### Nanoparticles accumulate in lymph nodes of live rats but not dead rats

**Figure 8:**
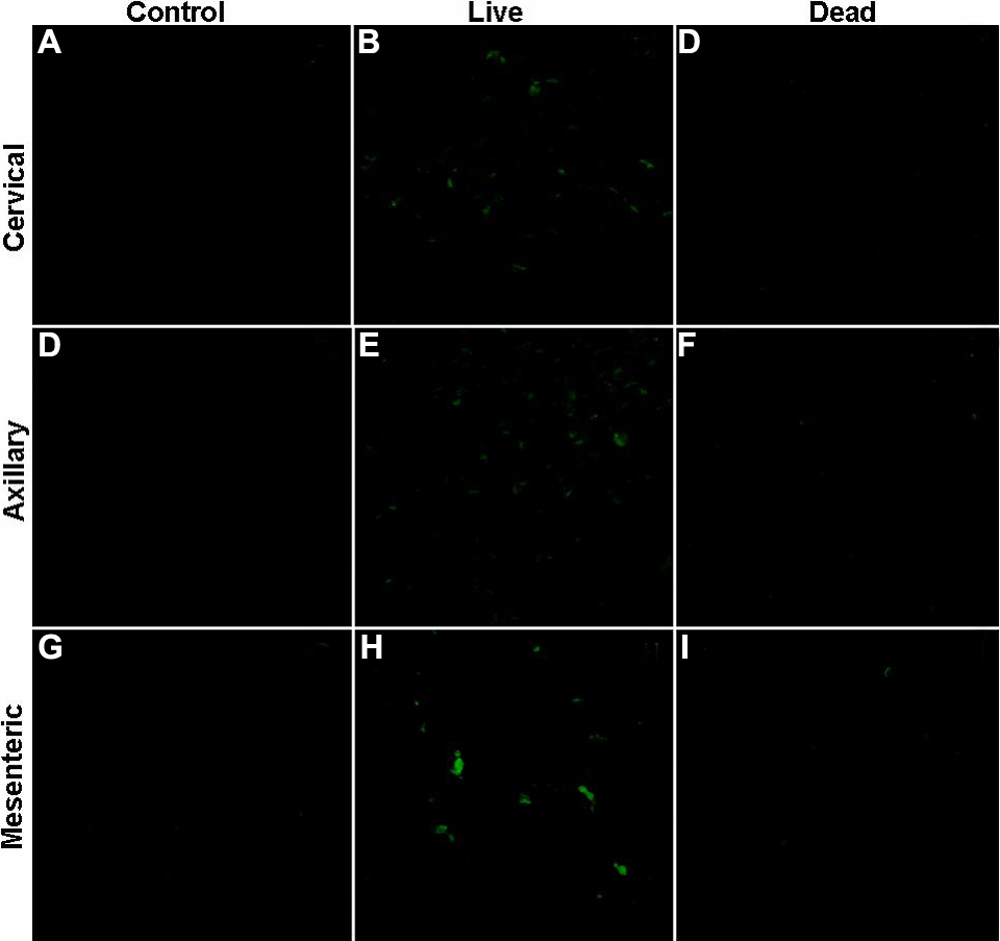
Representative confocal images of lymph nodes sections 6 h after periocular administration of 20 nm nanoparticles. Lymphatic circulation plays a role in the clearance of nanoparticles (20 nm) after periocular administration. Representative confocal images of lymph nodes sections, 6 h post periocular administration of 20 nm nanoparticles. Nanoparticles (20 nm; green) were administered to SD rats, live (Panels **B**, **E**, and **H**) and dead (Panels **C**, **F**, and **I**) by periocular injection. Lymph nodes, namely, cervical (Panels **A**-**C**), axillary (Panels **D**-**F**), and mesenteric (Panels **G**-**I**), were analyzed for the presence of nanoparticles by confocal microscopy. Lymph nodes of undosed SD rats were treated as controls (Panels **A**, **D**, and **G**). Green fluorescence associated with nanoparticles was observed in lymph node sections of live, but not dead, SD rats 6 h post periocular administration of nanoparticles. This suggests that in live animals lymphatic drainage delivered nanoparticles to various lymph nodes, however in dead rats, which are devoid viable lymphatic system, nanoparticles could not be detected in lymph nodes.

Nanoparticles were detected in all lymph node sections (cervical, axillary, and mesenteric) of live SD rats (Figure 8). On the other hand, no fluorescence was observed when rats were dosed with nanoparticles post-mortem. This observation suggests that lymphatic drainage might play a significant role in the clearance of 20 nm polystyrene nanoparticles from the subconjunctival site of injection.

### Simulated drug levels in the retina with 20 and 200 nm sustained release particles of celecoxib

**Figure 9:**
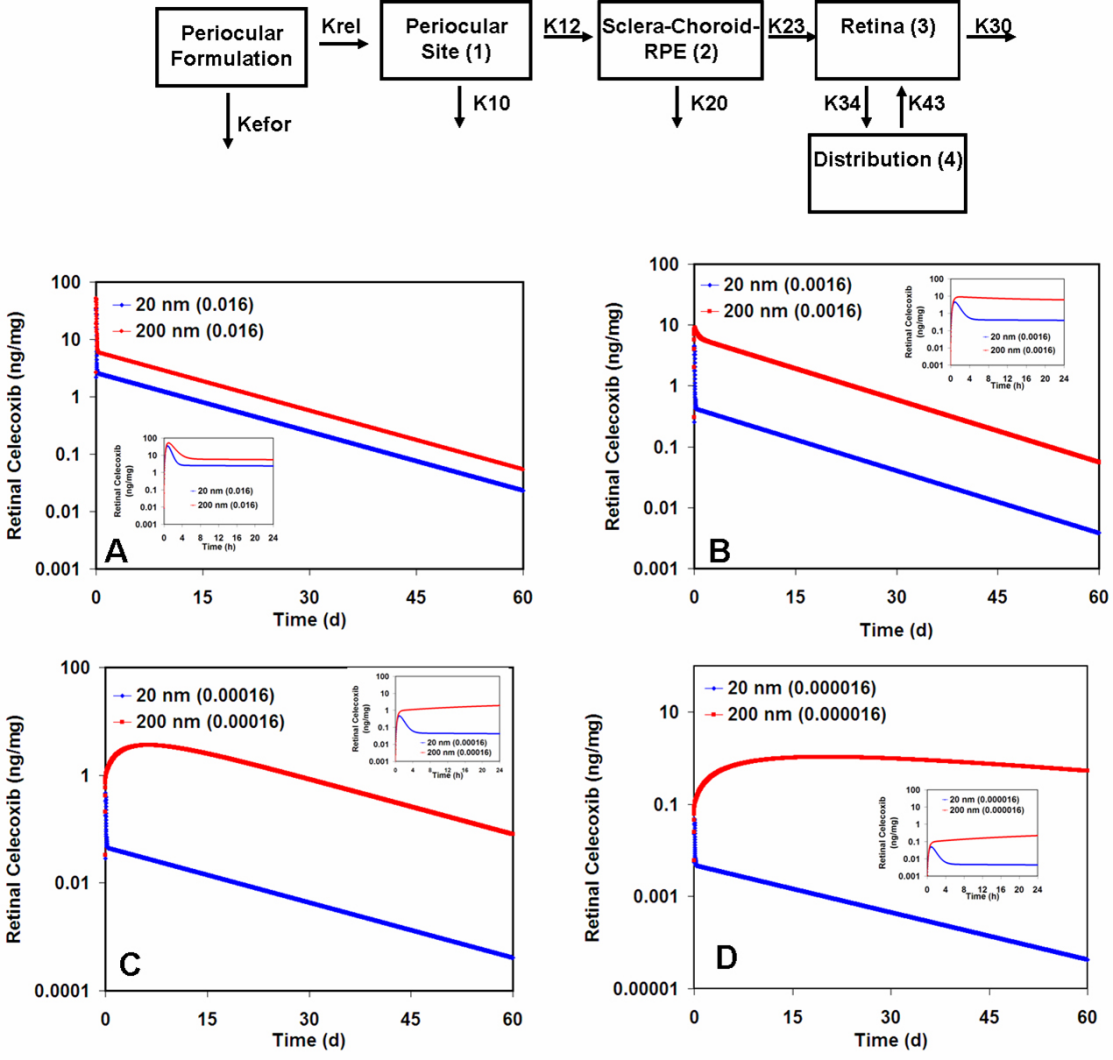
Sustained retinal delivery of a model drug (celecoxib) from nanoparticles with different clearance rates and drug release rates. The profiles were simulated for 20 nm and 200 nm particles for a period of 60 days. The elimination rate of the 20 nm formulation was obtained by curve fitting to the previously published data [12]. The estimated elimination half-life for 20 nm particles was 5.5 h. The elimination half-life for the 200 nm particles was assumed to be 180 days since they persisted almost completely for at least two months in the periocular space [12]. All other model parameters used in the model are shown in Table 1. The structural model is shown above the simulation. The panels depict profiles of 20 and 200 nm particles with a release rate constant of 0.016 min^−1^ (**A**), profiles of 20 and 200 nm particles with a release rate constant of 0.0016 min^−1 ^(**B**), profiles of 20 and 200 nm particles with a release rate constant of 0.00016 min^−1^ (**C**), and profiles of 20 and 200 nm particles with a release rate constant of 0.000016 min^−1^ (**D**). The insets in each panel are the profiles for the first 24 h of drug release to better show the early differences between the retinal concentrations of celecoxib using 20 and 200 nm particles.

We simulated the retinal drug levels over a 60-day time period in the retina following periocular administration of celecoxib sustained release particles (either 20 nm or 200 nm). The simulated profiles for the particles with different hypothetical first order release rate constants are shown in Figure 9. It is seen that the 200 nm particles better sustain retinal drug levels at all the release rates as compared to the 20 nm particles. Also, the difference between the drug levels with the 20 nm and 200 nm particles become more apparent when the drug release rates are lower.

## Discussion

In this study, we demonstrate that rapid clearance of 20 nm particles from the periocular space is due, at least in part, to the presence of blood and lymphatic circulations in the periocular region. Further, we demonstrate that 200 nm particles do not permeate bovine sclera or sclera-choroid-RPE over a 24 h period. However, 20 nm particles permeate across bovine sclera but not sclera-choroid-RPE. Further, our findings indicate that 200 nm particles do not clear rapidly from periocular space due to their poor movement across biological barriers. Due to size-dependent differences in the clearance of nanoparticles from the periocular space, differences in retinal delivery are to be anticipated for nanoparticles of different sizes even if the drug release rate is similar. Our simulations in this study suggest that larger nanoparticles (200 nm) better sustain the retinal drug delivery compared to smaller particles (20 nm) with the differences being the highest when the drug release is the slowest.

In a previous study, we found that there is a size dependent disposition of nanoparticles after periocular administration with the smaller particles (20 nm) rapidly cleared from the periocular tissue as compared to the larger particles (200 nm and 2 µm) [12]. We previously speculated that the rapid clearance of 20 nm particles was due to periocular blood or lymphatic circulation. In this study, we provide data to confirm this mechanism. The tissue levels of the particles in the sclera-choroid, which is the tissue most adjacent to the site of administration, are 19 fold higher in the dead animals when compared to the live animals. Since the periocular retention of the particles in the dead animals is only two fold higher, concentration gradient differences alone cannot explain the much higher levels of the particles observed in the sclera-choroid of the dead animals. The higher observed levels could be due to the absence of episcleral and/or choroid circulatory systems in addition to the absence of other periocular clearance mechanisms. The particle concentrations are higher in the retina and the vitreous (intraocular tissues) of dead animals as compared to the live animals. In live animals, the levels are below quantification limits in these tissues at several time points [12]. In addition to the absence of periocular and choroid clearance mechanisms in the dead rats, it is likely that the functional integrity of the RPE might have declined over time in these animals as compared to the live animals. Possibly due to a combination of reasons, including greater nanoparticle retention in the periocular site, greater nanoparticle entry/retention in the sclera-choroid, and the loss of integrity of RPE in the dead animals, we observed quantifiable levels of nanoparticles in the retina and vitreous of dead animals but not in live animals.

Particle levels are also higher in the cornea of the dead animals as compared to the live animals where the levels are below quantification limits. Drug transport to the cornea after periocular administration can partly be due to a leak-back through the needle track or through the conjunctiva into the tear film. It has been previously shown that there is a significant leak-back of the administered solution/suspension after periocular administration [19-21]. This leak-back can contribute to higher drug levels in the cornea at the initial time points. This has also been demonstrated using compartmental modeling of corneal pharmacokinetic data by Amrite et al. [15]. However, such leak-back might be more relevant for a drug in solution than nanoparticles, especially when there is no free drug present. A second explanation is that the entry of nanoparticles into the corneal epithelium might be less compared to previously tested drugs for which leak-back was suggested. This is supported by ex-vivo studies, which demonstrate that the corneal epithelium is a significant barrier to the entry of particles into the cornea [22]. A third explanation for the lack of quantifiable nanoparticle uptake in the cornea is that the assay for Fluospheres in tissues is not as sensitive as the previously used assays for small drug molecules. The higher corneal levels in dead animals at the end of 6 h could be partly due to the greater periocular space to tear film concentration gradient (two-fold higher) and partly because the elimination processes from the cornea are shut down. The elimination rate of drugs from the cornea is high as shown in several studies [23-26] including the modeling studies of Amrite et al. [15].

Using the data from the previous study [12] and an additional time point of 6 h in this study, we devised a simple pharmacokinetic model describing the disposition of the 20 nm particles from the periocular tissue. The disposition is biphasic and can be explained by a two-compartment model. There is a rapid clearance phase of the particles with a half-life of 5.5 h followed by a much slower elimination phase with a half-life of approximately 150 h. The 20 nm particles could be quantified on day 15 in the periocular tissue but not on day 60 [12] at which point, the levels were considered zero for half-life estimations.

Bourges et al. [27] demonstrated that large particles can move through the neural retina and reach the RPE after intravitreal administration. Bejjani et al. [28] demonstrated that plasmid-polymer particles administered intravitreally are able to transfect the retinal pigment epithelium, indicating their possible movement to these tissues from the vitreous. However, in the current study, we did not observe significant permeability for 200 nm particles across either sclera or sclera-choroid-RPE, indicating more formidable static barriers for transscleral nanoparticle delivery. Particles given intravenously are rapidly cleared from the circulation by the organs of the reticulo-endothelial system, specifically the liver and the spleen [29,30]. We observed detectable levels of particles in the spleen, using confocal microscopy after periocular administration of 20 nm particles. Further, a faint fluorescence signal was observed in the liver. Thus, particles enter the blood or lymphatic system from the periocular space and eventually reach the organs of reticulo-endothelial system. Geze et al. [29] demonstrated that following intravenous administration, the concentration of particles in the liver was much lower than in the spleen. In addition, we observed that the particles accumulated in the cervical, axillary, and the mesenteric lymph nodes following periocular administration in live animals. This lymphatic movement of particles has been demonstrated previously for latex nanoparticles [31]. It is also known that antigen presented to the eye can be carried to the lymph nodes as demonstrated in studies by Camelo et al. [32]. Thus, probably both the circulatory as well as the lymphatic systems play a role in periocular clearance of the 20 nm particles.

The particles used in this study are non-biodegradable, commercially available Fluospheres (Invitrogen, Carlsbad, CA). The particles are stable for prolonged periods of time in aqueous media and do not leach out any of the fluorescent material. Literature from the manufacturer indicates that there is less than 1% loss of the dye from Fluospheres after six months of storage in the dark in an aqueous medium and less than 10% change in the fluorescent signal in xylene after storage in the dark for six months. The Fluospheres as well as the dye are inert to alkaline hydrolysis when the temperature is maintained below 60 °C (Molecular Probes product information; FMRC manual). Thus, it is not very likely that the fluorescence detected in intraocular tissues is due to leaching of the dye from the Fluospheres.

The particles used in this study are negatively charged and have a relatively hydrophilic surface. Our previous studies indicated rapid, substantial clearance of 20 nm, hydrophilic particles as well as hydrophobic particles from the periocular site within a day unlike 200 nm and larger particles, which persisted in the periocular space for at least two months [12]. However, the retention of 20 nm, hydrophobic particles in the periocular space was greater compared to the hydrophilic ones. However, none of these particles entered the retina to any significant level. The reason for this, at least in part, is the poor permeability of nanoparticles across the sclera and more importantly across the sclera-choroid-RPE as evidenced in this study. It remains to be seen whether a change in surface property of nanoparticles including hydrophobicity and an incorporation of a positive charge alters sclera or sclera-choroid-RPE transport of 20 nm particles.

For sustained drug delivery, the delivery system should be retained at the site of administration. If there are mechanisms that lead to the clearance of the delivery system from the site of administration, they can have significant effects on the observed drug levels in the intended tissue. We simulated the drug levels in the retina after periocular administration of sustained release nanoparticulate systems of celecoxib, a model drug. We chose celecoxib because we had previous retinal pharmacokinetic data from periocular administration of celecoxib to rats [17,18]. We had also observed that delivery systems like celecoxib-poly(lactide-co-glycolide) particles can sustain in vitro release of celecoxib and deliver therapeutic levels of celecoxib to the retina [33,34]. In addition, our previous model, developed to describe the pharmacokinetics of small lipophilic molecules in the retina after periocular administration, was based on celecoxib [15]. In the previous model, the elimination of the pure drug suspension from the periocular tissue was considered to be negligible, and no elimination rate constant for the formulation was included. Based on the earlier study, we fixed tissue clearance properties for the drug. However, since small nanoparticles might be cleared more rapidly [12] and because polymeric particles can slow the drug release rates compared to what can be achieved with pure drug dissolution, we incorporated nanoparticle clearance from the site of administration into the model. We simulated the drug levels in the retina with different release rates for the delivery system. We modeled drug release with an initial drug release rate constant of 0.016 min^−1^, which is the in vivo dissolution or release rate constant (K_rel_) of celecoxib from pure drug suspension [15]. A sustained delivery system would have release rates significantly lower than this. Hence, we modeled releases from 20 nm and 200 nm particles with release rates over a four-order magnitude range. At all drug release rates, it is evident that the 200 nm particles best sustained the retinal drug delivery. The difference in retinal delivery between the 20 and 200 nm particles is larger at slower release rates. The 20 nm particles, due to their more rapid clearance, cannot sustain retinal drug delivery as well. In Figure 9, we compare retinal drug levels between 20 and 200 nm particles with same release rates in each panel. The release of a drug from a matrix system depends on the surface area of the particles. Hence, on a purely geometric basis, the release rate from a 200 nm sphere is expected to be 100 times less than the release rate from a 20 nm sphere of the same composition. Thus, the advantage of larger particles for sustained retinal drug delivery can be magnified when this factor is taken into consideration. Therefore, it is important to consider the elimination of particles from the site of administration in the design of a system for sustained drug delivery to the retina.

In summary, transscleral nanoparticle delivery is hindered by static as well as dynamic barriers. Larger nanoparticles persist in the periocular space due to their inability to move across both these barriers. Smaller nanoparticles with a diameter of about 20 nm, although capable of crossing static barriers such as the sclera to some extent, are rapidly cleared by the blood and/or lymphatic circulations and gain entry into organs of the reticulo-endothelial system such as the spleen and liver. Due to the rapid clearance of small nanoparticles and because of their inherent tendency to release the encapsulated drugs at faster rates, they fail to sustain retinal drug delivery as well as larger particles. Nanoparticle clearance or delivery system clearance from the site of administration in addition to the drug release rate is a critical factor in modeling drug delivery to the target site.

**Table 1:** Model parameters used for simulations of retinal pharmacokinetics after periocular administration of celecoxib in rats as particulate formulations with particle sizes, 20 and 200 nm

**Symbol**	**Meaning**	**Value (min^−1^)**
K10	Elimination rate constant from periocular site	0.123
K12	Absorption rate constant for sclera-choroid-RPE (transfer compartment)	3.61E-04
K20	Elimination rate constant from the sclera-choroid-RPE (transfer compartment)	0.035
K23	Absorption rate constant for retina from the sclera-choroid-RPE (transfer compartment)	0.061
K30	Elimination rate constant form the retina	0.002
K34	Rate constant for transfer to the distribution compartment from the retina	0.045
K43	Rate constant for transfer to the retina from the distribution compartment	0.001
Krel	Rate constant for the release of the drug from the formulation	Different values used
Kelfor	Rate constant for elimination of the formulation	Value chosen based on the formulation.

The structural model is shown in Figure 9. Except for the Krel and Kelfor, all other parameters were chosen from a previous study [15].
